# Visceral leishmaniasis in a patient with diabetes mellitus type 2 and discrete bicytopenia

**DOI:** 10.1002/ccr3.1259

**Published:** 2017-11-28

**Authors:** Verena Schwetz, Christian Trummer, Claudia Friedl, Christine Beham‐Schmid, Roman Kulnik, Albert Wölfler, Karl Horvath, Stefanie Wunsch, Jürgen Prattes, Ines Zollner‐Schwetz, Thomas R. Pieber, Julia K. Mader, Robert Krause

**Affiliations:** ^1^ Division of Endocrinology and Diabetology Department of Internal Medicine Medical University of Graz Auenbruggerplatz 15 8036 Graz Austria; ^2^ Division of Nephrology Department of Internal Medicine Medical University of Graz Auenbruggerplatz 15 8036 Graz Austria; ^3^ Department of Pathology Medical University of Graz Neue Stiftingtalstraße 6 8010 Graz Austria; ^4^ Division of Nuclear Medicine Department of Radiology Medical University of Graz Auenbruggerplatz 9 8036 Graz Austria; ^5^ Division of Haematology Department of Internal Medicine Medical University of Graz Auenbruggerplatz 15 8036 Graz Austria; ^6^ Department of Internal Medicine Section of Infectious Diseases and Tropical Medicine Medical University of Graz Auenbruggerplatz 15 8036 Graz Austria

**Keywords:** Leishmania, pancytopenia, splenomegaly, visceral leishmaniasis

## Abstract

An Austrian patient with diabetes mellitus type 2 developed visceral leishmaniasis after trips to Spain and Crete, presenting with slight bicytopenia, later developing severe pancytopenia. Travel history taking is important due to an extended incubation period. Coexistence of diabetes mellitus can impair T lymphocyte function and cause higher relapse rates.

## Introduction

Visceral leishmaniasis (VL) is a protozoan infection by *Leishmania donovani* or *infantum* transmitted by sandflies. The annual global incidence is half a million cases in endemic zones 1 including the Mediterranean countries 2. Tourism and HIV infection are associated with imported VL 3. Therefore, thorough travel history taking is crucial.

Weeks to months after a trip to endemic regions, fever, loss of appetite, weight loss, lymphadenopathy may occur, often accompanied by pancytopenia, hepatosplenomegaly, and elevated liver enzymes 4, 5. The infection may mimic leukemia, viral infections, or autoimmune diseases. By polyclonal B‐cell activation, multiple positive serologic tests can appear 6, 7, 8.

Diagnostic approaches include histopathology, in vitro culture, molecular detection of parasite DNA –the most sensitive assay –and serologic testing. Bone marrow aspiration is the preferred sample source; liver, lymph nodes, and whole blood are also possible 9. Treatment with liposomal amphotericin B is recommended 9.

This report features a case of VL with only discrete bicytopenia, as pancytopenia only developed gradually, adding to a delay in diagnosis together with equivocal positive serologic tests (elevated levels of the soluble IL2‐receptor) and a travel history to Greece and Spain 12 and 4 months earlier, thus expanding our knowledge on diagnostic pitfalls of VL.

## Case Report, Differential Diagnoses, Investigations, Treatment, Follow‐up

In September 2016, a 60‐year‐old male Austrian patient presented at the Department of Internal Medicine, Medical University of Graz, Graz, Austria, with a three‐week history of fever of unknown origin, fatigue, and unintentional weight loss of 6 kg. Initial physical examination was inconspicuous. Concomitant diseases were adequately treated diabetes mellitus type 2 and hypothyroidism. Travel history revealed a two‐week trip to the south of Crete in September 2015 and a two‐week trip the south of Spain in May 2016.

Laboratory parameters showed elevated C‐reactive protein (CRP) ranging between 94.2 and 188.9 mg/L (normal range 0–5 mg/L), slight hypochromic, normocytic anemia without signs of hemolysis (hemoglobin 12.5 g/dL), mild thrombopenia (127,000/*μ*L; normal range 140,000–440,000/*μ*L) and a discreetly elevated gamma‐glutamyltransferase (GGT) but were otherwise inconspicuous. The soluble interleukin 2‐receptor (IL‐2R) was elevated (5156.1 pg/mL, reference range 458.0‐1997.0). Chest X‐ray, urine analysis, abdominal sonography, transthoracic as well as transoesophageal echocardiography showed no focus of infection but revealed splenomegaly (7.2 × 16.4 cm). Serial blood cultures were negative, as were tests for influenza (negative PCR), other viral infections (negative IgM antibody test for Puumala, Dobrava, and Hantaan virus, negative PCR for cytomegalovirus and Epstein–Barr virus), leptospirosis (negative antibody test), malaria (negative blood smear), tuberculosis (negative interferon‐gamma release assay), and human immunodeficiency virus (HIV) (negative antibody).

Empiric antibiotic therapy with initially ampicillin/sulbactam and later piperacillin/tazobactam plus vancomycin did not lead to improvement. Interestingly, in the course of the following 3 weeks, discrete bicytopenia turned into severe pancytopenia requiring blood transfusions. Lactate dehydrogenase (LDH), initially normal, increased to 1107 U/L (reference range 120–240). Liver enzymes including GGT, alkaline phosphatase (AP), aspartate transaminase (AST) also increased. Fever persisted with levels up to 40°C.

**Figure 1:**
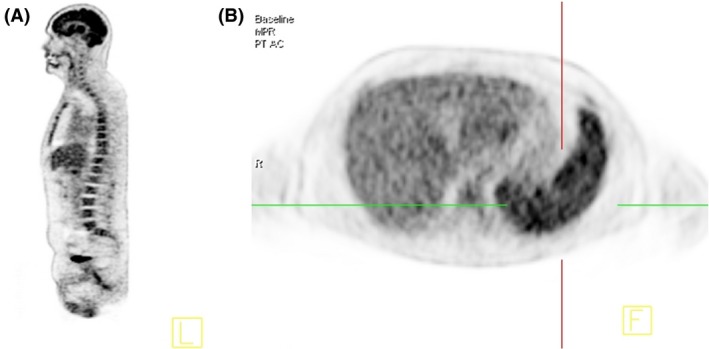
(A) Homogeneously increased 18F‐FDG‐uptake in the bone marrow. (B) Homogeneously increased 18F‐FDG‐uptake in the spleen. PET/CT scan was performed using a dedicated 64‐slice body PET/CT scanner (Biograph mCT). A 6‐h fasting period is required before the intravenous injection of the activity of 356 MBq 18F‐FDG can be applied. Imaging was started with a low‐dose CT of the whole body 1 h after the injection. PET scans were performed in caudocranial direction with 2 min per bed position.

**Figure 2:**
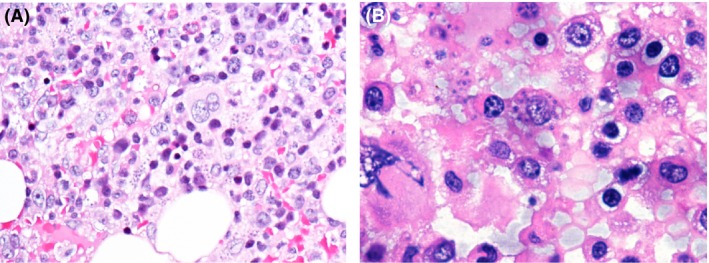
(A and B) Pathological examination of the bone marrow including hematoxylin and eosin (H&E) staining revealed macrophages with intracytoplasmic microorganisms.

Positron emission tomography–computed tomography (see Fig. 1A and B) showed tracer enhancement in the central and peripheral bone marrow. Bone marrow biopsy revealed microorganisms within macrophages (Fig. 2). PCR from peripheral blood and bone marrow confirmed infection with *Leishmania donovani*. Therapy with liposomal amphotericin B was initiated, causing rapid cessation of fever and a significant drop in CRP levels within days. Blood count ameliorated rapidly, and transfusions were no longer required. After release from hospital, regular follow‐up visits at the outpatient clinic ensued. Until today, VL has not recurred.

## Discussion

In the patient reported, the positive IL‐2R test complicated diagnosis of VL, as did the presumed early stage of VL featured in the discrete bicytopenia. In the course of the disease, severe pancytopenia developed and liver enzymes gradually rose (AP, GGT, AST). In the literature, prevalence of bicytopenia, leukopenia, and thrombocytopenia were reported to be 41.7%, 61.1% 10 and 64–83.3% 10, 11, respectively, of pancytopenia between 43% and 75% 10, 11, 12. Frequency and severity of pancytopenia thus vary, and pancytopenia is mostly seen after prolonged duration of illness due to splenic sequestration of blood cells 13, complicating diagnosis in early stages.

Fever in VL is almost always present 10, 11, splenomegaly in 23.7% to 100% of patients 10, 11, elevated liver enzymes in 42–85% of cases 5, 11, increased levels of LDH and CRP are seen in 72.5% and 83.1% of cases 11. In the patient presented here, CRP was elevated from the first day of presentation and remained unchanged until initiation of therapy, LDH started rising with the appearance of pancytopenia.

Although uncommon in travelers, VL needs to be considered in patients with unexplained febrile illness, especially when hepatosplenomegaly and thrombocytopenia are present and a positive travel history to an endemic area is reported 4. Travel history should be traced back to months or even years, as VL has a very long incubation period up to several years 4, 14. The long time from infection to disease onset is a fact that might often be elusive to the general internist rarely confronted with VL.

Diagnosis of VL may further be complicated as the infection can mimic more common diseases such as hematologic malignancies, viral infections, or also autoimmune diseases due to polyclonal B‐cell activation causing positivity of many serologic tests 7, 8. In the patient presented here, elevated levels of the soluble IL2‐receptor (sIL‐2R) were found without further evidence of sarcoidosis, possibly owed to polyclonal B‐cell activation in visceral leishmaniasis. sIL‐2R has previously been suggested as a marker of disease severity in VL, as it is high at the beginning of infection and returns to normal following successful antibiotic therapy 15.

Any kind of immunocompromising disease may be a predisposing factor of VL as shown by two‐thirds of the patients described in Fletcher's cohort 14 who were either HIV positive or had autoimmune diseases. Of the nine immunocompromised patients who relapsed, two were found to have either diabetes mellitus or chronic alcohol abuse, both diseases potentially impacting on T lymphocyte function 16, 17, 18. This might be especially crucial in VL, as T cells are critical in controlling leishmania protozoa. The patient presented had diabetes mellitus type 2, a potential predisposition for the development of VL 19. Indeed, prevalence of diabetes mellitus was reported to be 13% in a Greek cohort 5. As the risk of relapsing might also be elevated, regular follow‐up visits are performed. In the period of follow‐up until now, no relapse has occurred.

## Conclusion

Diagnosis of VL in travelers in nonendemic countries is challenging, as it is a rare disease mimicking more common hematological, viral, or even autoimmune diseases. Detailed travel history is crucial, as VL has a very long incubation period of up to several years. Immunocompromised patients with HIV, autoimmune diseases, chronic alcohol abuse, or diabetes mellitus are at a higher risk of VL than immunocompetent individuals and may face lower cure and higher relapse rates.

## Authorship

VS: wrote the manuscript. CT, CF, KH, TRP, JM: were involved in patient care and conception of the manuscript. CBS: was involved in establishing the diagnosis from a pathological point of view and in conception of the manuscript. RK: was involved in diagnostic work‐up and conception of the manuscript. AW: was involved in patient care from a hematological point of view and in conception of the manuscript. SW, JP, IZS and RK: were involved in patient care from the Section of Infectious Diseases and in conception of the manuscript.

## Conflict of Interests

Verena Schwetz, Christian Trummer, Claudia Friedl, Christine Beham‐Schmid, Roman Kulnik, Albert Wölfler, Karl Horvath, Stefanie Wunsch, Ines Zollner‐Schwetz, Thomas R. Pieber, Julia K. Mader, and Robert Krause have no conflict of interests. Jürgen Prattes received consulting fee from Gilead.
